# Supplementary material for: Stabilization of Pickering Emulsions by Hairy Nanoparticles Bearing Polyanions
Source: Polymers (Basel). 2019 May 7;11(5):816. doi: 10.3390/polym11050816 (PMC6571738; doi:10.3390/polym11050816)
Supplement: Supplementary file 1 [file polymers-11-00816-s001.pdf]

Stabilization of Pickering Emulsions by Hairy Nanoparticles Bearing Polyanions

Ying Zhang [1], Kaimin Chen [2,*], Lan Cao [1], Kai Li [2], Qiaoling Wang [2], Enyu Fu [2] and Xuhong Guo [1,*]

[1] State Key Laboratory of Chemical Engineering, School of Chemical Engineering, East China University of Science and Technology, Shanghai 200237, China; zy12fearless@163.com (Y.Z.); lancaoecust@outlook.com (L.C.)

[2] College of Chemistry and Chemical Engineering, Shanghai University of Engineering Science, Shanghai 201620, China; 18301939658@163.com (K.L.); woodsues@outlook.com (Q.W.); fuenyu1234@163.com (E.F.)

***** Correspondence: kmchen@sues.edu.cn (K.C.); guoxuhong@ecust.edu.cn (X.G.);
Tel.: +86-137-0172-0354 (K.C.); +86-137-6436-8083 (X.G.)

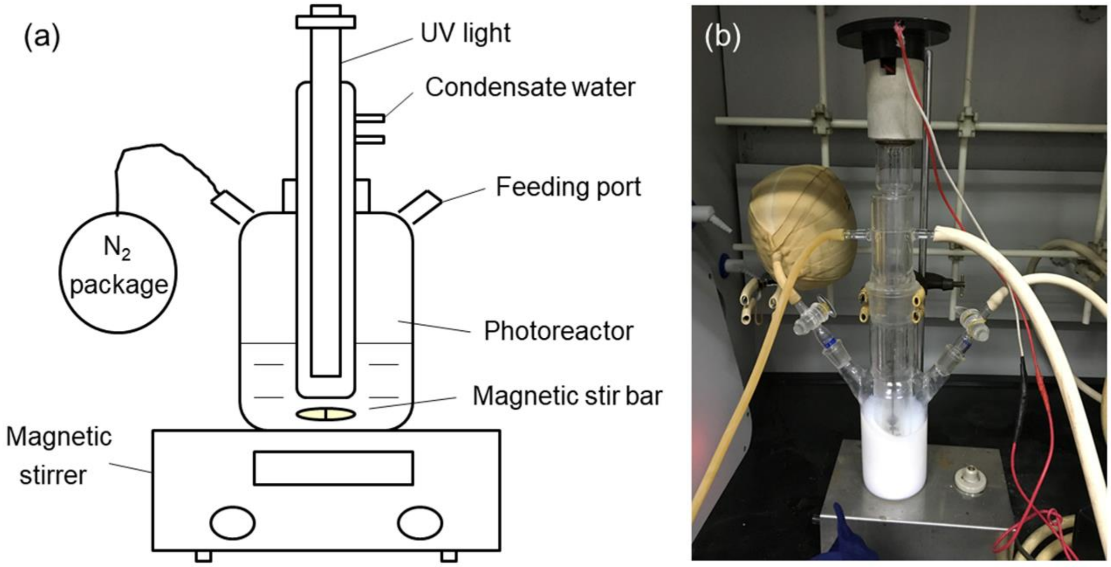

Figure S1. Schematic diagram (a) and photo (b) of the home-made photoreactor

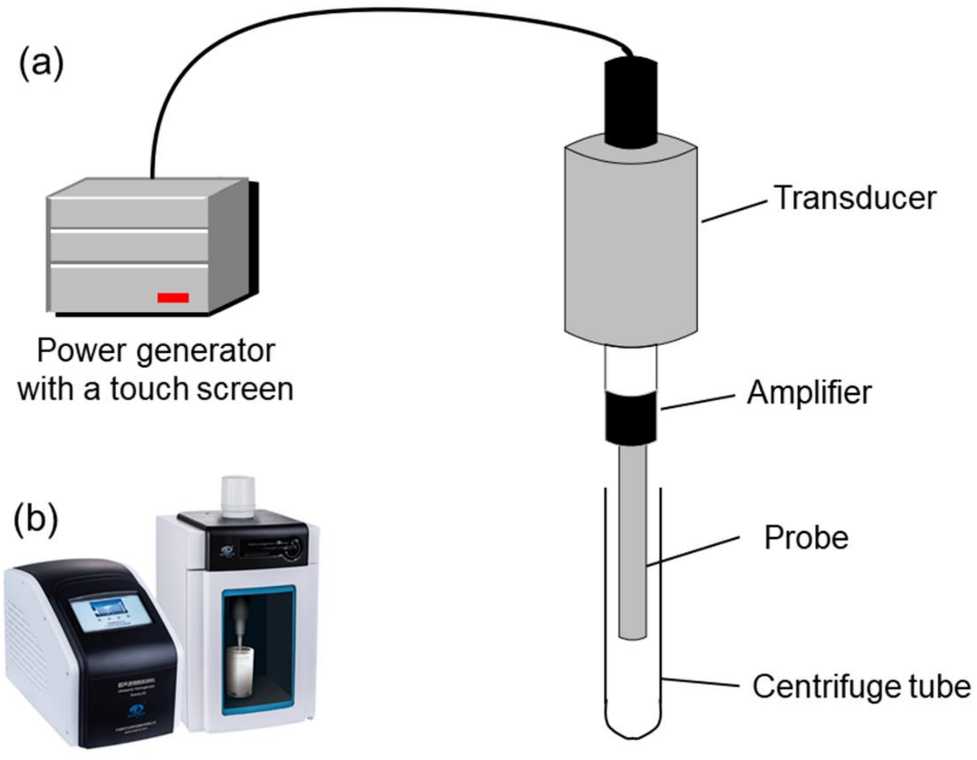

Figure S2. Schematic diagram (a) and photo (b) of ultrasonic homogenizer

Table S1. The effect of pH on PS@PSS size.

pH	PS@PSS diameter (nm)	Thickness (nm)	PDI
4	250.3±0.6	88.1±0.4	0.035±0.012
5	249.4±1.3	87.6±0.8	0.027±0.004
6	253.7±0.3	89.8±0.3	0.043±0.019
7	248.9±0.8	87.4±0.5	0.021±0.015
8	252.5±1.9	89.2±1.1	0.027±0.011
9	252.9±0.8	89.4±0.5	0.016±0.007
10	250.3±0.5	88.1±0.4	0.024±0.014
11	249.1±1.7	87.5±1.0	0.044±0.023

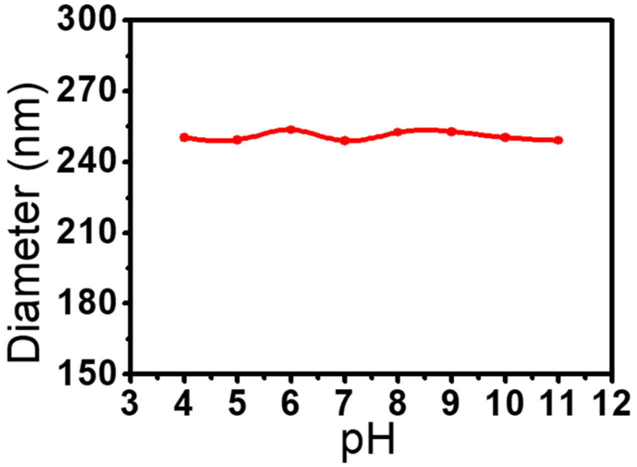

Figure S3. The effect of pH on PS@PSS size.

No changes of PS@PSS diameter with pH was observed. So PS@PSS nanoparticles are not responsive to pH.
